# P-960. Improving Resident Education on the Inpatient Infectious Disease Consult Service

**DOI:** 10.1093/ofid/ofae631.1150

**Published:** 2025-01-29

**Authors:** John Flores, Anna Czapar, Michael Z Chen, Palak Patel, Eric Roessler, Christopher Kaperak, Sabrina Imam, Elizabeth Bell, Christopher Lehmann, Daniel Z P Friedman, Aniruddha Hazra

**Affiliations:** University of Chicago Hospital, Chicago, Illinois; University of Chicago Medicine, Chicago, Illinois; University of Chicago, Chicago, IL; University of Chicago, Chicago, IL; University of Chicago, Chicago, IL; University of Chicago Medicine, Chicago, Illinois; University of Chicago, Chicago, IL; University of Chicago Medicine, Chicago, Illinois; University of Chicago, Chicago, IL; University of Chicago, Chicago, IL; University of Chicago, Chicago, IL

## Abstract

**Background:**

Education in infectious disease (ID) is a critical part of an internal medicine curriculum. As the infectious disease consult service is typically high volume and busy, residents and other trainees were often not receiving adequate training in less common infectious conditions or didactic training in common conditions that may be relevant for their future practice or internal medicine board exams. Our Quality Improvement project sought to improve training for residents and medical students through the integration of high-yield lecture services given on the Adult ID consult service.

**Figure 1:**
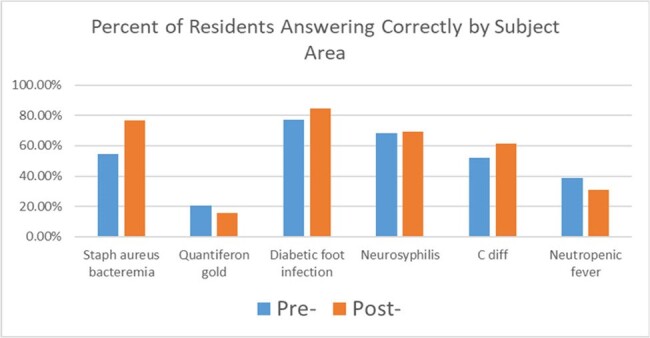
Pre/Post Test Results of Internal Medicine Residents

These are the reported correct responses, in terms of percentage of residents who responded correctly to the case-based multiple choice question, to a pre and post test of six specific high-yield topics in Infectious Diseases.

**Methods:**

During the academic year of 2022-2023, for the Adult ID Consult Service, a series of high-yield lectures were given three times a week from a set of 10 potential lecture topics, as chosen by the volunteer faculty or ID fellow lecturer. Following our first Plan-Do-Study-Act cycle evaluation in July 2023, the lectures were standardized to a fixed rotating schedule of the 6 highest-yield topics. There was distributed a survey with a pre-test before the rotation, and a follow-up survey and post-test following the rotation.

Post-intervention Interest in Career in ID among Internal Medicine Residents

**Figure:**
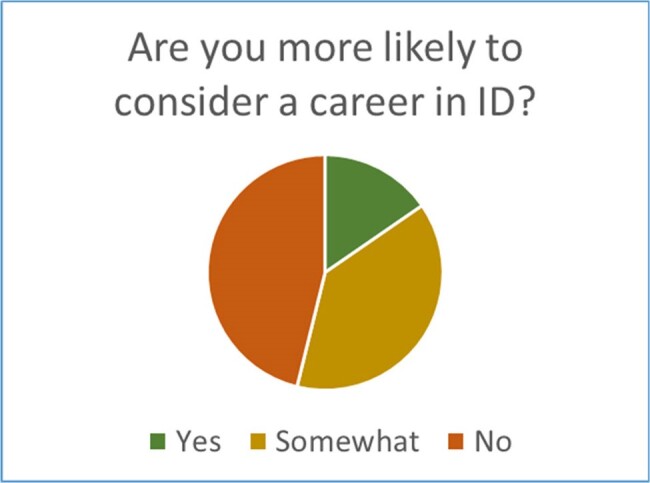

15.4% (n=2) of internal medicine residents responded yes they would, and 38.5% (n=5) responded somewhat more likely, to consider a career in Infectious Diseases (ID) following the rotation.

**Results:**

44 Internal Medicine Residents took the initial Adult ID survey and pretest, and 13 people took the post-rotation evaluation survey and post-test. 100% of participants felt the rotation fully or somewhat helped prepare for the Adult ID section of Internal Medicine boards. 92.3% (n=12) felt the majority of cases on rotation to be interesting. 15.4% (n=2) responded yes and 38.5% (n=5) responded somewhat more likely to consider ID as a career. 100% (n=13) of post-test participants responded with strongly agree (n=7, 53.8%) or agree (n=6, 46.2%) on enjoying the Adult ID rotation. Additionally, scores on the post-test improved from the pre-test for most residents, along with an improvement in Internal Medicine in-training exam scores from 2021 to 2023.

**Conclusion:**

A didactic series with a set lesson plan that is delivered in a low-stress way can be helpful for engaging learners, enhance the educational experience of the learner, help supplement preparation for internal medicine board exams, and prepare the learner for their future practice. Additionally, as there is a growing need for infectious diseases fellows, this may improve recruitment efforts.

**Disclosures:**

**Aniruddha Hazra, MD**, Gilead Sciences: Advisor/Consultant|Gilead Sciences: Grant/Research Support|ViiV Healthcare: Advisor/Consultant

